# A simplified protocol for Agrobacterium-mediated transformation of cell suspension cultures of the model species *Medicago truncatula* A17

**DOI:** 10.1007/s11240-023-02495-6

**Published:** 2023-03-23

**Authors:** Ana Clara Ferreira, Bárbara A. Rebelo, Rita Abranches

**Affiliations:** Plant Cell Biology Laboratory, Instituto de Tecnologia Química e Biológica António Xavier, ITQB NOVA – Universidade Nova de Lisboa, Av. República, 2780-157 Oeiras, Portugal

**Keywords:** Model legume, Medicago, Cultured cells, Plant-based system, Agrobacterium, Stable transformation, SARS-CoV-2, RBD

## Abstract

This manuscript describes a unique protocol for the rapid transformation of *Medicago truncatula* A17 cell suspension cultures mediated by *Agrobacterium tumefaciens*. Medicago cells were collected on day 7 of the growth curve, which corresponded to the beginning of the exponential phase. They were then co-cultured with Agrobacterium for 3 days before being spread onto a petri dish with appropriate antibiotic selection. The Receptor Binding Domain of the Spike protein of SARS-CoV-2 was used as a model to develop this protocol. The presence of the transgene was assessed using PCR, and the integrity of the product was evaluated by SDS-PAGE and Western-blotting.

## Key message

We have developed an effective and simple protocol for the transformation of *Medicago truncatula* A17. The success of this protocol relies on proper handling of the cultures and is based solely on co-cultivating plant cells with Agrobacterium. This method does not require experienced professionals or specialized equipment, making it faster, easier, and more affordable than other available protocols. Additionally, there is no need for protoplast preparation or vacuum application.

## Introduction

Plant cell suspension cultures are a valuable tool in numerous areas of plant research, serving as models for both fundamental cell biology studies and more applied biotechnology, such as recombinant protein production. Effective transformation protocols for cell suspension cultures are essential for various areas, namely the availability of rapid processes that allow a swift answer to the problem under study. Each plant species presents its own specificities, particularly the higher or lower tendency to form aggregates in liquid culture, making it challenging to implement standardized transformation protocols.

The widely used tobacco BY-2 cell culture line was the first to be transformed by Agrobacterium, during the 1980’s (An 1985). Since then, many other commonly used species have been transformed by similar methods, such as suspension cultures of tomato (McCormick et al. 1986), rice (Baba et al. 1986), soybean (Baldes et al. 1987), carrot (Wurtele and Bulka 1989) and grapevine (Martínez et al. 2015). However, these procedures often involved a first step of protoplast preparation, which is both laborious and time consuming. There are not many reports of straightforward transformation protocols and the ones available often require many steps, including multiple washing procedures that increase the chance for unwanted contamination.

*Medicago truncatula* cell cultures are highly versatile and utilized for a variety of purposes. The standard genotype for this species is A17 which has been fully sequenced (Young et al. 2011) and displays a wide array of available genetic tools. Compared with the widely used tobacco BY-2 cell line, *Medicago truncatula* cell cultures have significantly lower proteolytic content (Santos et al. 2018), making them an attractive option for the production of recombinant proteins and other applications.

In this report, we introduce a simple and rapid method, named suspension culture-based (SB) protocol, which allows the generation of transformed liquid cultures in about 12 weeks. Importantly, this method is suitable for less experienced researchers and does not require specialized equipment, such as a desiccator or vacuum pump. Furthermore, it is more economical and involves fewer steps than Santos et al. (2019), hereinafter referred to as *calli*-based (CB) protocol, making it easier to maintain sterility throughout the process and avoid possible contamination. Finally, it can be easily adapted to other plant species.

## Materials and methods

### Biological material

Cell suspension cultures of *M. truncatula* cv. Jemalong line A17 were generated from seeds following the procedure by Sello et al. (2017) and maintained as outlined below:

1. Medicago seeds were scarified with concentrated sulfuric acid (10’) and washed 5 times with sterile distilled water. The seeds were sterilized by immersion in a solution of 5% (V/V) commercial bleach with 0.012% (V/V) Tween 20 for 5 min. Subsequently, they were washed with sterile distilled water, immersed in 70% (V/V) ethanol (2’), washed again with sterile distilled water, and let dry on a sterile Whatman filter paper (Kondorosi and Denarié 2001).
2. Approximately 15 seeds were placed onto petri dishes containing Murashige and Skoog medium including vitamins [Duchefa] supplemented with 30 g/L sucrose [Duchefa], 0.25 mg/L 6-benzylaminopurine [BAP, Duchefa], 0.5 mg/L 2,4-dichlorophenoxyacetic acid [2,4-D, Sigma-Aldrich], pH 5.5, 0.7% micro agar [Duchefa] and maintained at 23 °C with an 12/12 h light/dark cycle for 2–3 weeks.
3. *Calli* formed at the hypocotyl level were transferred to petri dishes containing CIM medium, prepared as follows: Gamborg B5 medium including vitamins [Duchefa] supplemented with 20 g/L sucrose, 0.1 mg/L BAP, 1 mg/L 2,4-D, 5 mg/L citric acid [VWR], 5 mg/L ascorbic acid [Sigma-Aldrich] and 0.7% micro agar. The pH of the medium was adjusted to 5.5 with KOH.
4. Five to ten *calli* of approximately 1 cm of diameter were dispersed in 20 mL of liquid CIM medium and grown in 100 mL erlenmeyers in the dark, on a rotary shaker at 23 °C, 110 rpm [Agitorb 200IC, Aralab]. A17 cells were subcultured every 10 days, by adding 5 mL of inoculum to 20 mL of fresh medium.

### Agrobacterium tumefaciens growth (3–4 days)

In this study we used the Receptor Binding Domain (RBD) of the SARS-CoV-2 coronavirus sequence codon optimized for *Nicotiana tabacum* and cloned into pTRA vector (pTRA_RBD) as described in Rebelo et al. (2022).

1. *A. tumefaciens* GV3101::PMP90RK containing pTRA_RBD was grown in YEB medium supplemented with 2 mM Mg_2_SO_4_, 50 mg/L Rifampicin and Kanamycin and 80 mg/L Carbenicillin (antibiotics from NZYTech).
2. Bacteria were incubated in the dark at 28 °C, 200 rpm, for 2–3 days, and subcultured onto the same medium afterwards. Critical Step (CS): bacterial overgrowth can lead to reduced transformation efficiency.
3. After 1 day, Agrobacterium cells were harvested by centrifugation (2300 g × 5’), resuspended in infiltration medium (Murashige and Skoog medium [Duchefa], 50 g/L sucrose, 2 g/L glucose, pH 5.3, supplemented with 200 µM acetosyringone [Sigma-Aldrich] (Rademacher et al. 2019)) to an optical density (OD_600nm_) of 0.5, 0.6 or 1. The bacterial suspension was incubated for 2 h at room temperature (RT) before infiltration.

### Transformation of the Medicago liquid culture (about 12 weeks until obtaining stable positive liquid cultures)

**Fig. 1:**
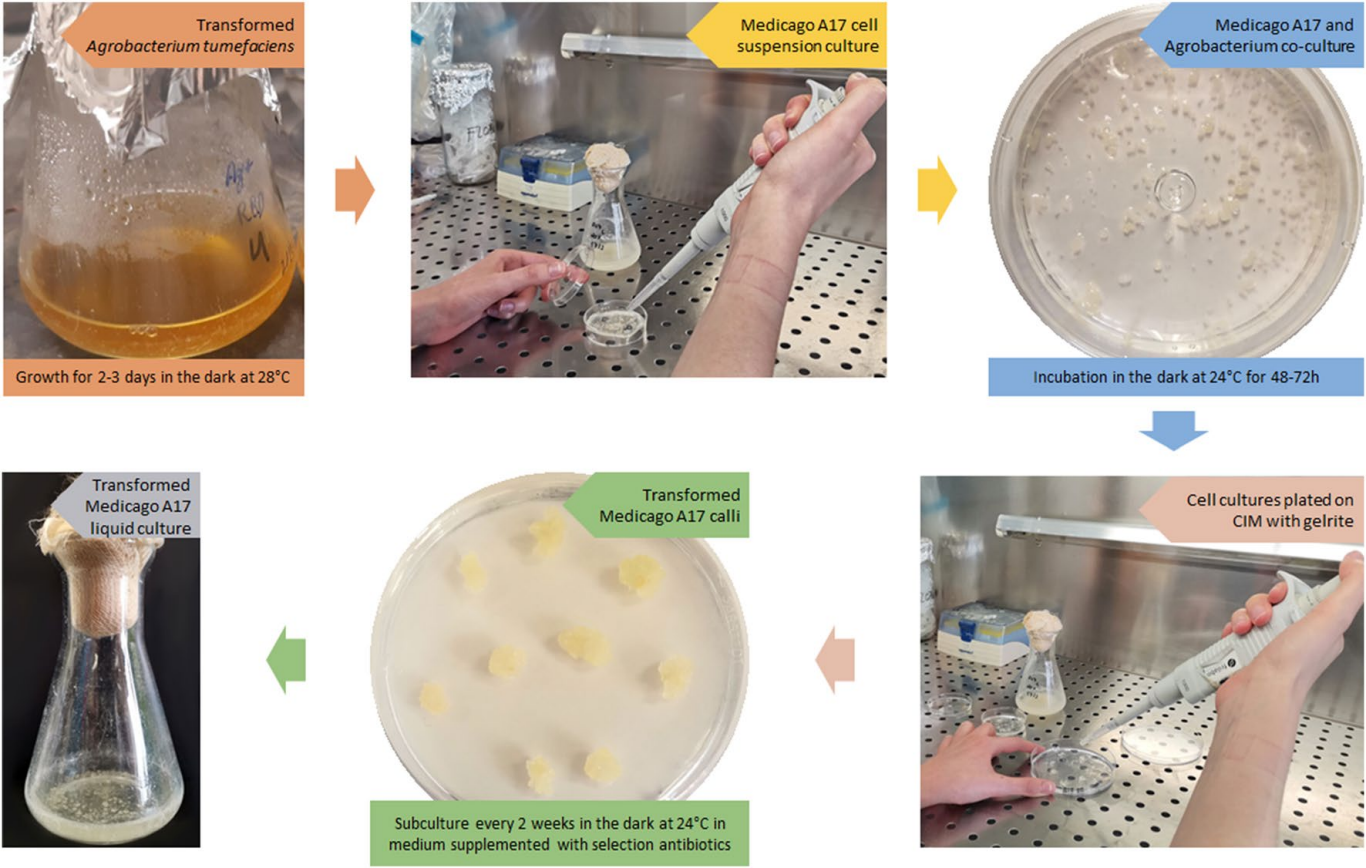
Schematic representation outlining the main steps of the suspension culture (SB) transformation protocol of Medicago cultured cells

Step-by-step illustrated guide of Medicago transformation with SB protocol is depicted in Fig. 1.

1. At day 7 of growth, 4 mL of Medicago liquid culture were transferred to three petri dishes (55 mm Ø). Then, 100 µL of Agrobacterium suspension with a final OD_600nm_ of 0.5, 0.6 or 1 were added to each petri dish and gently mixed. CS: use wide bore tips or cut at least 1 cm off normal tips for transferring Medicago cells.
2. The petri dish was sealed with cling film and incubated at 24 °C in the dark for 60–72 h.
3. 4 mL of CIM medium were added to the co-cultures and spread onto petri dishes (92 mm Ø) prepared with CIM medium, 0.4% (w/V) Gelrite [Duchefa], 500 mg/L ticarcillin disodium/clavulanate potassium [Timentin, Duchefa] for Agrobacterium elimination and 100 mg/L Kanamycin for transformant selection.
4. The petri dishes were placed in the dark at RT until the first *calli* appeared. CS: Growing micro-*calli* were isolated and subcultured every 2 weeks, to fresh plates containing the selection antibiotic and a 50% stepwise decrease of Timentin concentration.
5. After three rounds of selection they were moved to liquid culture by placing a fragment of the *calli* in liquid CIM medium supplemented with Kanamycin and dispersed with the help of a sterile blade or disposable loop. Medicago suspension cultures were subcultured every 10–15 days to fresh medium with 20% inoculum.
6. In parallel, the transformation of Medicago *calli* was carried out following the CB protocol (Santos et al. 2019).

### Assessment of the RBD gene presence in transgenic Medicago lines

1. At day 10 of growth, Medicago transgenic or wild-type cultures were paper-filtered, cells were collected and macerated in liquid nitrogen, using a mortar and pestle.
2. DNA was extracted using the NZY Plant/Fungi gDNA Isolation Kit [NZYTech], following the manufacturer’s instructions.
3. Quality and integrity of the extracted DNA was assessed by agarose gel electrophoresis and spectrophotometric analysis.
4. PCR amplification was performed to the extracted DNA samples, following the GoTaq® DNA Polymerase [Promega] supplier instructions and with the following primers 5’-ATCCTTCGCAAGACCCTTCCTCT-3’ and 5’-AGAGAGAGATAGATTTGTAGAGA-3’.
5. PCR products were separated by agarose gel electrophoresis to evaluate gene amplification.

### Protein extraction and Western blot analysis

1. At day 10 of growth, Medicago cells and spent medium were collected by filtration.
2. Medicago *calli* with 4 weeks and collected cells were macerated in liquid nitrogen, using a mortar and pestle.
3. Per each gram of *calli* or cells, 1 mL of extraction buffer (100 mM Tris-base, 10 mM ascorbic acid, pH 7.5) or 2 mL of extraction buffer (10 mM Ascorbic Acid, 500 mM NaCl, 5 mM β-mercaptoethanol, pH 8) were added, respectively, and the extracts incubated on ice for 1 h.
4. The extracts were centrifuged (16,000 g × 15’, 4 °C) and the supernatant was kept at -20 °C.
5. 4 × sample buffer (320 mM Tris-base pH 6.8, 0.04% bromophenol blue, 8% SDS, 40% glycerol, 20% β-mercaptoethanol) was added to the cell extracts (1:3 V/V) and the samples were boiled at 100 °C for 10 min.
6. Spent medium samples were precipitated using ice-cold ethanol (sample to ethanol ratio 1:4), and resuspended in 1 × sample buffer, with a final concentration of tenfold.
7. Samples were loaded onto 12.5% SDS-PAGE polyacrylamide gels and the proteins were blotted onto a nitrocellulose membrane using Trans-blot Turbo Transfer System [Bio-Rad].
8. The membranes were incubated in a blocking solution (5% (w/V) skimmed milk and 3% (w/V) bovine serum albumin in phosphate-buffered saline containing 0.1% (V/V) Tween 20 (PBS-T)) for 1 h at RT.
9. The membranes were washed with PBS-T (3 × 5’) and incubated with anti-RBD (1:2000) [40592-T62, SinoBiological] for 1 h at RT and then at 4 °C o/n.
10. The membranes were washed with PBS-T (3 × 5’) prior to and following incubation with secondary antibodies HRP conjugated anti-rabbit (1:10,000) [AS09 602, Agrisera] or StarBright Blue 700 anti-rabbit IgG (1:20,000) [12004162, Bio-Rad] for 2 h at RT.
11. Protein signal was detected by chemiluminescence or fluorescence in iBright™ FL1500 Imaging System [Invitrogen™].

### Quantification of RBD protein in Medicago culture medium

1. Spent medium samples (A17 WT, CB1, CB2, CB3, SB6, SB7 and SB9) were concentrated fivefold, as described in the previous sub-section.
2. The samples were resolved in 12.5% SDS-PAGE polyacrylamide gels, and the proteins were stained using BlueSafe Reagent [NZYTech].
3. A standard curve with BSA at concentrations of 2, 3, 5, 10 and 20 mg/L was built and used to determine the relative amount of secreted RBD.
4. Recombinant RBD protein bands were quantified using Image Lab Software v6.1 [Bio-Rad].

## Results

**Fig. 2:**
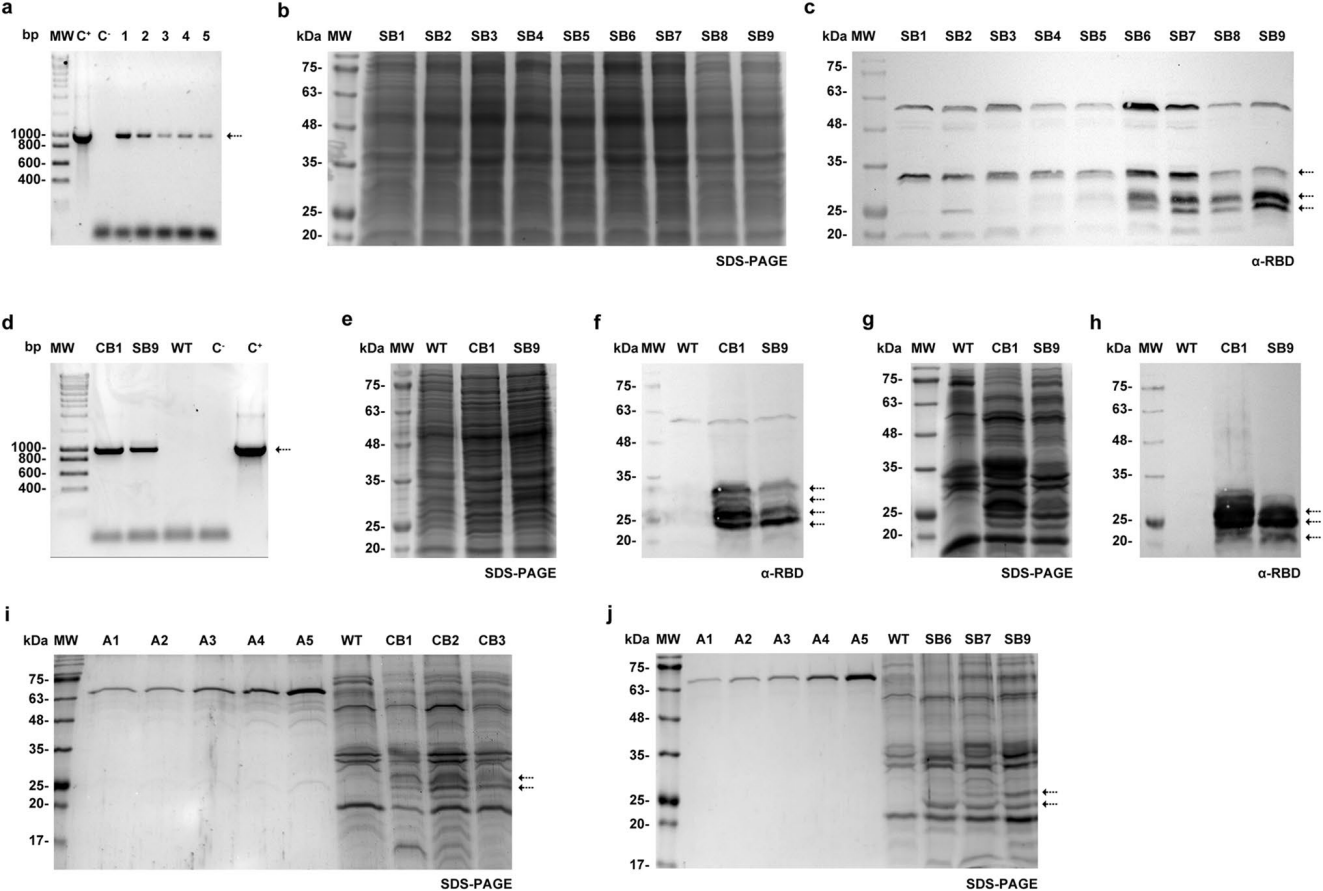
Characterization of transgenic Medicago cell lines obtained with two different transformation protocols. **a** Colony PCR for the detection of the RBD gene in five Agrobacterium colonies using primers for the 35S promoter and terminator regions. **b**, **c** SDS-PAGE stained gel of Medicago *calli* (**b**) and Western-blotting with anti-RBD antibody (**c**). **d** PCR analysis of gDNA of two transgenic Medicago lines (CB1 and SB9) for RBD detection. **e**–**h** Detection of RBD protein by SDS-PAGE stained gels and Western-blotting with anti-RBD antibody in cell protein extract (**e**, **f**) and spent medium (**g**, **h**). **i**, **j** Quantification of the secreted RBD protein in three independent transgenic lines (CB1-CB3) obtained with the CB protocol and three independent transgenic lines (SB7, SB8, SB9) obtained with the SB protocol. MW: NZYDNA Ladder III (**a**, **d**), NZYColour Protein Marker II [NZYTech] (**b**, **c**, **e**–**j**); C^+^: pTRA_RBD vector; C^−^: PCR negative control (water); WT: wild-type; Medicago transgenic lines transformed with Agrobacterium OD_600_ 0.5 (SB1-SB3), OD_600_ 0.6 (SB4-SB5) and OD_600_ 1 (SB6-SB9); A1-A5: Loading of 80, 120, 200, 400 and 800 ng of BSA corresponding to 2, 3, 5, 10 and 20 mg/L, respectively

Medicago *truncatula* A17 transgenic lines were generated using two different methodologies. The first one is a *calli*-based protocol previously developed in our laboratory (Santos et al. 2019). The second method is a shorter, simpler Agrobacterium-mediated transformation protocol resulting from the combination of previously described procedures (An 1985; Rademacher et al. 2019). Medicago liquid cultures were used instead of *calli*, which reduced the time needed to obtain a sufficient number of cells for transformation. Agrobacterium containing the pTRA_RBD vector, confirmed by colony PCR (Fig. 2a), was incubated with acetosyringone to induce virulence genes and co-cultivated with Medicago cells for 3 days. After co-cultivation, micro-*calli* were grown on solid CIM media, with proper antibiotic selection, and subcultured every 2–4 weeks onto the same medium to maintain continuous production.

Our goal was to establish a fast and straightforward transformation protocol. To assess the success of the new protocol, we used the RBD protein as a model. First, we evaluated the RBD production in *calli* obtained with the SB protocol, where bacterial suspensions with varying OD_600nm_ were tested (Fig. 2b, c). All three ODs tested were found to be very efficacious, yielding numerous micro-*calli* that grew under antibiotic selection. Western blot analysis with anti-RBD antibody detected several bands, corresponding to the non-glycosylated RBD (25 kDa) and putative glycosylated forms (around 35 kDa). This pattern showed some variation among lines; however, this analysis was performed on *calli* which are clusters of cells that may be heterogeneous in terms of total soluble protein (TSP). After establishing liquid cultures using both protocols, we extracted genomic DNA from one line of each protocol (CB1 and SB9) to confirm the presence and integrity of the transgene, as shown in Fig. 2d, using specific primers for the 35S promoter and terminator respectively. We recommend that this assay is carried out prior to the selection of Medicago transgenic lines when there is no specific antibody available to detect the recombinant product, as this will save time in the screening of the putative transformed lines. Western blot analysis was performed on total protein extract (Fig. 2e, f) and spent medium (Fig. 2g, h). All RBD isoforms were detected, as previously shown in Fig. 2c, except for the higher molecular weight glycoform of RBD which was not present in the spent medium. Quantification of the secreted RBD was performed relative to a BSA standard curve, using normalized samples for TSP or volume. We recommend that quantifications are performed in view of the specific objective of the work; for a biotechnological application, it is more important to determine the amount of recombinant product that is obtained per volume of culture, regardless of the number of cells or the total protein found per liter of culture. In this study, we did not evaluate a sufficient number of cell lines generated by each protocol to perform statistical analysis, but we were able to determine that the amount of secreted RDB in lines originating with the *calli*-based method was more heterogeneous ranging from 0.5 to 2.8 mg/L while the suspension culture-based protocol yielded around 1.6 mg/L, as depicted in Fig. 2i, j. Recombinant RBD protein is secreted to the culture medium of Medicago, from which it can be purified, reinforcing the positive outcome of applying this optimized transformation protocol*.* Our results demonstrated that both protocols are suitable to generate Medicago A17 cell lines producing and secreting the model protein.

## Discussion

In this work, we report an optimized transformation protocol for Medicago cell suspension cultures that requires only a flow chamber to maintain sterility, with no other specialized equipment needed. Our previous transformation protocol for *M. truncatula* cells (described in Santos et al. 2019) required the plant material to be under vacuum to promote the entry of Agrobacterium in Medicago cells. If not properly carried out, the transformation would not be effective. With this new protocol, we have mitigated this critical step since the co-culture of plant cells with the bacterial suspension is sufficient to carry out Agrobacterium gene transfer. Although the previous transformation protocol (Santos et al. 2019) is efficient, it involves several steps that carry a higher risk of contamination. The *calli* must be filtered and two additional steps using filter paper are necessary before transferring them to solid CIM medium. Importantly, this protocol requires using *calli* as starting material, which can take up to four weeks to reach the proper size, thereby extending the overall procedure time. There is a protocol for the generation of transgenic plants of *M. truncatula* cv. Jemalong 2HA which employs liquid cultures and the procedure is similar to our protocol (Iantcheva et al. 2014; Iantcheva and Revalska 2019). In this work, Medicago cells were co-cultured with Agrobacterium and positive transformants were screened based on GFP or GUS activity. However, their goal was the regeneration of fertile plants and not the establishment of liquid cultures. Other reported protocols for the transformation of liquid cultures of other species include additional steps such as protoplast preparation, removal of bacterial cells by washing steps, or placing the cells onto a sterile paper prior to the subculture in the solid medium. These procedures increase the difficulty to transfer the cells in an individualized way (e.g. Wu et al. 1998; Moniruzzaman et al. 2021; Badim et al. 2022).

To validate the effectiveness of the new transformation protocol, we selected a recombinant protein with a high impact in current worldwide research, the Receptor Binding Domain of the SARS-CoV-2 virus (Rebelo et al. 2022). Other ongoing projects in the laboratory, in which we have used this new protocol, indicate that it is faster and simpler than previous methods (Rebelo et al. in preparation; Vieira et al. in preparation). The vector containing the RBD gene was used in parallel for transformation of Medicago cell cultures using the two methods developed in our lab. The quantification of the secreted recombinant protein showed no relevant differences between the transgenic lines obtained by the two protocols, but we did not evaluate a sufficient number of lines to apply statistical analysis. Furthermore, we did not assess the number of copies inserted in each transgenic line or the site of transgene integration in the host genome. These features would be interesting to investigate in a broader population of transformed cell lines, in particular, it would be important to assess if the use of suspension cells vs *calli* as starting material impacts on the number of transformation events. Within the small sample that we evaluated, we detected a higher heterogeneity among lines derived from the *calli* method, with respect to the amount of recombinant product found in the spent culture. This could mean that more independent transformation events took place, which can ultimately result in less predictability of the cells´ behavior in terms of production. Further studies are necessary to assess this possibility, and this will be useful to further improve transformation protocols for plant cells, independently of the species under study.

The methodology presented in this report is a straightforward Agrobacterium-mediated transformation process that may be implemented for other plant cell suspension cultures of different species to rapidly obtain transgenic cultures.
